# Rural Height Penalty or Socioeconomic Penalization? The Nutritional Inequality in Backward Spain

**DOI:** 10.3390/ijerph18094483

**Published:** 2021-04-23

**Authors:** Antonio M. Linares-Luján, Francisco M. Parejo-Moruno

**Affiliations:** Faculty of Economics and Business, University de Extremadura, Avda. Elvas s/n, 06010 Badajoz, Spain; fmparejo@unex.es

**Keywords:** Extremadura, height, nutrition, anthropometric indicators, inequality, nutritional health, biological well-being, living standards

## Abstract

This article studies the evolution of nutritional inequality, measured through the male adult height, in one of the poorest regions of Spain, in southwestern Europe: Extremadura. With a wide sample of statures of recruits born between 1855 and 1979, conscripted between 1876 and 2000, the research delves into the urban-rural height gap using coefficients of variation, tests of equality of means and proxy variables of a socioeconomic nature. The results of the analysis reveal that the strong anthropometric growth that Extremadura experienced since the last decades of the 19th century was accompanied by a less internal inequality. The lower heterogeneity did not eliminate, however, the urban-rural height gap during the period under study. In this sense, despite the absence of environmental differences between urban and rural areas in Extremadura, there was a clear rural height penalty in the region from the mid-19th century to the late 20th century. Rural punishment was fundamentally related to the greater presence of agrarian workers and the lower presence of wealthy families in villages and small towns. On the contrary, educational differences or differences in terms of nutritional health were not as decisive in the rural height penalization, at least when such differences are measured with the sources of military recruitment.

## 1. Introduction

The ability of adult height to capture the influence of different factors during childhood and adolescence, in addition to the genetic factor, has made this measure a proxy variable for the biological well-being [1,2,3]. The anthropometric literature maintains that the stature reached at the end of the physical growth stage (20–22 years) is the expression of the “net nutritional status”: what remains of the nutrition after discounting the energy consumed by the basal metabolism, the physical effort and disease [4]. From this perspective, stature has become a good indicator of health, nutrition and, in general, of biological standard of living.

In the same way, and since adult height synthesizes the socioeconomic conditions in which physical development occurs from birth [5,6], stature has proven to be also a good measurement of inequality. Height differences by education level, place of residence, social status, professional occupation, family wealth, labor market or among members of different castes o ethnic groups, have been used to analyze inequality in various parts of the world from diverse perspectives [7,8,9,10]. Likewise, the variability of stature has also made it possible to identify the impact of the institutional framework on inequality [11,12]. From this point of view, the role of the State as provider of public goods, the structure of land ownership, or the composition of households have become in explanatory factors of the disparity of statures and, therefore, of the differences in the biological well-being [13,14].

Within the distinct determinants of stature inequality, the place of residence and, more specifically, the height gap between urban and rural areas has been one of the facts that has received most attention. This difference has been accompanied by a hypothesis that is not without controversy, the existence of an “urban height penalty” because of the industrialization process. The hypothesis of such punishment seems to be particularly true when applied to the first countries in which industrialization took place, the so-called “first comers”. For these territories, there is an extensive bibliography showing that in the early stages of the contemporary economic modernization the combination of industrialization and rapid population growth resulted in an urban height penalty and, by contrast, in a “rural height premium” [15,16,17].

Several reasons can explain why the stature of urban dwellers decreased relative to rural statures in the early phases of industrialization [18]. The growth of cities in the heat of the development of factories worsened living and working conditions in the urban world. New industrial workers did not receive significant increases in real wages or clear improvements in food consumption. In addition, industrialized cities tended to be overcrowded and to suffer from pollution, overcrowding and sanitation problems, which could increase disease transmission. Considering that rural life traditionally offered nutritional advantages due to proximity to agriculture [8], stagnation of purchasing power for factory workers and worst working conditions, coupled with an infection-prone environment, kept stature in urban areas below that of the rural ones, at least for much of the XIX century in the first industrialized countries.

In them, the urban height penalty declined over time and finally the rural premium reversed into a penalization [18]. The improvement of transportations and market integration increased the supply of foods in cities, making the former rural advantages in nutrition less evident. The public investment in water supply and sanitation improved health conditions in the urban areas [19]. Medical services and sanitary innovations also tended to expand more rapidly in cities and large towns than in rural world [8]. As a result, during the last decades of the 19th century the difference between urban and rural stature shortened or was reversed [18]. In fact, from the early 20th century onwards, as a rule, urban dwellers became taller than their rural contemporaries [20].

This general account of an urban height penalization during the 19th century becoming in a rural height penalty in the 20th century is, nevertheless, far from universal [18]. Historical experience of some regions reveals not only the existence of processes opposed to the one previously described, but also the absence of an urban height penalization during the 19th century [16]. This last is the case, among others, of France [21], Switzerland [22], Württemberg, Bavaria, and Prussia in Germany [23,24], the eastern region of Belgium [25], the northern Italy [26], Lisbon in Portugal [27], or Catalonia [18], Castile-Leon [28], and the southeast of Castile-La Mancha in Spain [29]. In the same way, there are also evidence showing a rural height advantage, both in Europe and in the USA, during the 19th and 20th century [30,31,32,33].

In such circumstances, the only thing that seems clear is that, since the Industrial Revolution, there has not been a unique and irreversible evolutionary line in the urban-rural height gap. Nor is there currently a single explanation for the differences that anthropometric historiography has observed in this regard between territories. From the different peculiarities that urban and rural areas acquire in each country or in each region, to the various institutional conditions under which these areas develop, there are many variables that can explain anthropometric gap between the countryside and the city during the last centuries.

**Figure 1:**
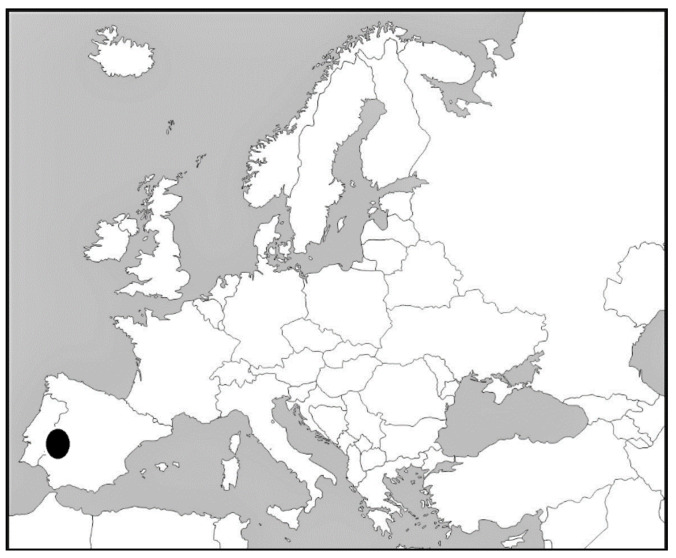
Extremadura in Europe.

It is just here, in the geographical diversity that height inequality shows throughout contemporary history, where the present research fits. In it we analyze the case of Extremadura, an extensive Spanish region, located in the southwest of the country, bordering with Portugal (Figure 1). Unlike most of the territories studied in Spain, this region has never been industrialized, not even during the so-called “Spanish developmentalism” of the 1960s. Consequently, or as a cause of it [34], Extremadura has not been in the last centuries characterized by the existence of big, unhealthy, or overcrowded cities. If to this is added a very low productivity rainfed agriculture, dominated by the large exploitation and by concentration of land ownership, the region under investigation becomes a magnificent laboratory to test the hypothesis of the existence (or absence) of an urban-rural gap in terms of biological well-being. As, moreover, the analysis is carried out on a truly big dataset (138,231 records) and for a really long period of time (1855–2000), we think that the results of our research are truthfully significant for anthropometric historiography.

These results reveal that, once the so-called *crisis agraria finisecular* (end-of-the-century agrarian crisis) was overcome, the biological living standard in Extremadura, measured through adult stature, began a process of sustained growth that was only slightly interrupted during the 1920s. Such a “leap forward” was accompanied in the region by a progressive reduction in inequality that, however, did not eliminate the anthropometric gap between countryside and city. In this sense, despite the limited environmental differences between urban and rural areas in Extremadura, it was “rural height penalty” that dominated the region throughout the period under study. Indeed, it was precisely the rural world that suffered most intensely the crises that the evolution of stature in the region showed from the mid-19th century to the end of the 20th century. The reasons for this punishment are not easy to weigh with the available sources. However, from the tests carried out here, two factors emerge as the most important determinants of the urban-rural height gap in Extremadura: the economic (professional) difference and the social ones. Other variables of an educational or nutritional health nature are analyzed in our study, but none of them is conclusive when it comes to explaining rural height penalization.

## 2. Materials and Methods

The height data used in this research come from the so-called *Actas de Clasificación y Declaración de Soldados* (henceforth ACDSs) and are the result of the Military Recruitment Law of 1856. With some exception, corrected in 1912, this law established compulsory male military service in Spain and obligation to collect in each town hall of the country the medical and anthropometric information of all recruits called up at each conscription. Since then, until 2001, every year, around the same dates, in each Spanish municipality all young men of legal enlistment age were called for the official recognition. From these annual calls the ACDSs were born. In them, not only the stature is recorded, but also the thoracic perimeter and, since 1955, the weight of each recruit. In addition, they include the filiation, the place of birth and of residence, the level of literacy, and, though not always, the occupation and the economic situation of the conscript’s family. All this qualitative information is very useful to carry out disaggregated analyzes, such as the one proposed here.

The sample used to this article, which we have called Extremadura’35, comes from the men conscripted between 1876 and 2000, born between 1855 and 1979, in thirty-five Extremadura’s localities. Some of these municipalities fit perfectly into what demographers call “agro-cities” [35], large towns with a significant proportion of agrarian population but with a greater representation of the industry and services than in rural nuclei. The remaining population entities follow the guidelines observed by Extremadura’s economic historiography for the region as a whole [36]: absolute preponderance of the agrarian sector until well into the 1950s, little representation of the manufacturing industry, even from 1960, and growing importance of the construction and services since the 1920s.

In quantitative terms, the magnitude of the sample drawn from the ACDSs gives statistical robustness to our analysis. Of the 138,231 records gathered for the conscripts born between 1855 and 1979, almost 87% (120,078) contains accurate information on the height of each recruit. The remaining 13% is composed mainly of individuals who were declared “fugitives” or “alleged fugitives”; those who, residing in municipalities other than that of recognition, were classified as “pending” due to lack of data; the sick youth who were hospitalized or were “in bed”; and those other young men who, while fit for military service, did not attend the act of recognition because they were serving as “volunteers” in the army.

In summary, the sources used for this study collect the results of a universal male conscription that only leaves out of each generation the previous cases of exclusion. It is to say, the average annual height of the conscripts recruited in the thirty-five localities of the sample is also, except for error or omission, the same as that of all the men who, having been born in such entities, reached the legal age of military enrolment. This facet of the ACDSs is statistically important. In some countries, stature data often stem from institutions that imposed a height requirement, minimum and maximum, as a precondition of entrance [37]. Obviously, the samples extracted from these records are not representative since they are really truncated samples. Spanish military legislation also imposed a minimum size for military service (156 cm: henceforth cm), but all youth of enlistment age were included in the ACDSs, regardless of whether they were finally classified as unfit by local authorities. Thus, there are no truncation problems in the samples extracted from the ACDSs, although, to work with them in a diachronic way, it is necessary to correct the differences in stature that the changes in the age of enlistment generate [38].

In Spain, such age was mostly 21 years, just when experts consider that the stage of physical growth in the male population has concluded. However, this age was modified several times throughout the period under study. Extremadura conscripts, for example, were measured at 18 years old in 1996–2000, at 19 in 1885–1899 and 1991–1995, and at age 20 in 1878–1884, 1901–1905 and 1986–1990. Given that in contexts exposed to nutritional stress physical development could exceed the 20-year-old threshold, the sample Extremadura’35, like those from the rest of the country, must be corrected standardizing at 21 years the mean height of the recruits conscripted to the 18–20 years.

There are different methods of standardization [28,39,40]. The one used here [39] consists of calculating the 50th percentile (P_50_) for the conscripts between the ages of 18 and 21 and, afterwards, add the resulting differences between ones and the others to height of the recruits conscripted before age 21. Previously, however, given that throughout the period under study the average stature of the young men conscripted in Extremadura increased significantly, we have calculated the height differences for two distinct generations: the generation born between 1855 and 1890, conscripted between 1876 and 1911, and the generation born between 1954 and 1982, conscripted between 1975 and 2000 (Table 1). Thus, for generation 1855–1890, 1.8 cm has been added to the stature of the conscripts of 19 years age, and 0.9 cm to those of 20 years. For generation 1954–1982, the stature of men recruited at 18 has been increased by 1.7 cm, that of those measured to the 19 by 0.9 cm, and that of those of 20 years old by 0.3 cm.

Starting from standardized data and in order to delve into the urban-rural height gap, we have constructed four time series of statures, using the arithmetic mean as the centrality statistic. To smooth out the variability of stature in the short term, the recruits in our sample have been divided into 5-year consecutive groups (cohorts). Based on them, the first series offers the number of valid cases that each cohort comprises, and the mean stature of all recruits born between 1855 and 1979, distinguishing between standardized and non-standardized heights.

**Figure 2:**
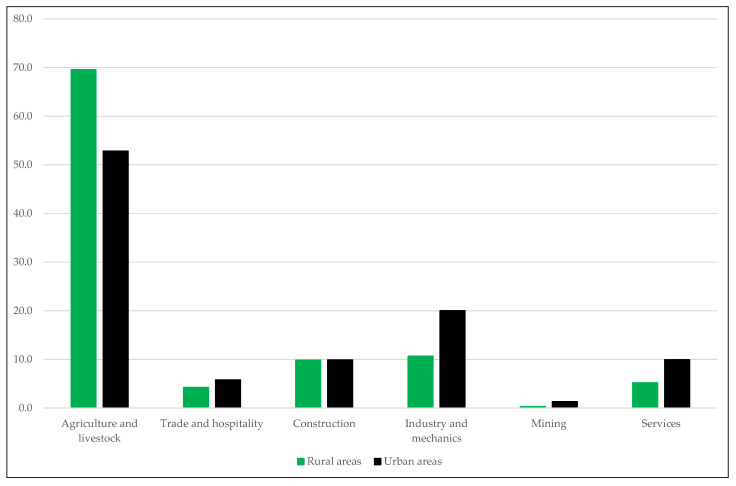
Sectoral distribution of the occupations declared by the recruits included in the sample Extremadura (percentage of cases in each sector over the total records with information on occupation).

In the second series, we have segmented our sample by place of residence, making the distinction between urban and rural areas. The criterion used to distinguish one area from other has been the size registered in the Population Census of Spain of 1920 [41], an intermediate year between the beginning and the end of the period under study. According to this census, we have assigned the urban category to nuclei with more than 10,000 inhabitants (cities and large towns) and the rural category to those with less than 10,000 inhabitants (villages and small towns). This criterion may be debatable [42,43], but it is the most one used by Spanish historical demography and by anthropometric historiography. In the case of Extremadura, in addition, the division seems consistent with the particularities that agro-cities offer at the regional level. Thus, according to the occupations that the recruits of our sample themselves declare to exercise (Figure 2), urban areas present an economic structure that is skewed towards the primary sector, but with a more important representation of the industry and services than in rural areas.

Our third series of statures attempts identify the determinants of the urban-rural height gap, disaggregating the sample Extremadura’35 by professional occupation. To not complicate the analysis, we have distinguished only between agrarian workers and non-agrarian workers. The hypothesis behind this simple classification is that, in poor areas with low nutrient consumption, like Extremadura, the physical effort required by the agricultural and livestock tasks, at least until the mechanization that brought with in the so-called “green revolution” since the mid-1950s, may have generated shorter statures in agrarian workers than in non-agrarian ones.

The following series of heights uses the professional information provided by the ACDSs to distinguish between recruits who declare to be students and the rest of recruits who claim to exercise a particular occupation. When we speak of students, we are referring specifically to young people who at the time of enlistment are pursuing university studies or, at the very least, secondary education. From this perspective, the social component of the new variable is evident. Not in vain studying a university career in Spain until well into the 20th century has been a difficult luxury to assume for most Spanish families. This fact makes it possible to identify students among the social groups with the highest income and, therefore, with a best access to nutrition, hygiene, and health. It is not by chance that all research carried out so far in Spain coincide in pointing to the students as the tallest of all the conscripts recruited in the course of the 19th and 20th centuries [44].

We complement our four anthropometric series with four more time series constructed from the number of records contained in the sample Extremadura’35. The first one shows for each area, urban and rural, the percentage of the recruits who declare working in agrarian sector over the total number of recruits who report on their profession, except those who claim to be students. The objective in this case is to evaluate the extent to which rural areas in Extremadura concentrate a higher proportion of agrarian workers than urban areas. If the difference in proportions between one area and other is maintained over time and if the difference in statures between agrarian and non-agrarian workers is also maintained throughout the period under study, it seems clear that professional activity may be one of the explanatory keys to the urban-rural height gap.

The same is true of our second series, disaggregated by place of residence: the proportion of recruits who report being students over the total number of conscripts who declare to exercise a profession. The objective in this case is to try to know to what extent the anthropometric superiority that the specialized literature attributes to the students with respect to the rest of the conscripted population allows us to explain the urban-rural height gap in Extremadura.

In the third time series, we distinguish between those recruits who claim to know how to read and write and those who confess not knowing to do it. This educational information does not allow us to know the actual level of literacy achieved at 21 years of age. In fact, some studies cast doubt on the veracity of the opinion of the conscripts and conclude that the functional literacy of the Spanish recruits was generally lower than that indicated in the sources of military recruitment [45]. We think, nevertheless, that such information can illuminate our analysis not only from the perspective of the conscripted population, but also from the possibility of detecting from it differences in access to education between the recruits’ families. With such objectives, we have calculated for urban and rural areas an illiteracy rate expressed as the proportion of recruits who declare not being able to read or write over the total number of recruits for whom our sources offer information on literacy. This time the purpose is related to the conclusions of a previous research [46]. According to it, in Extremadura the literate recruits were always taller than the non-literate ones. If this is the case and the illiteracy rates in rural areas differ markedly from those in urban areas throughout the period under study, we could be facing another of the possible explanatory keys for the urban-rural height gap. But not only from an educational perspective. As some studies reveal [47,48,49,50] literacy can also be interpreted as an alternative measure of social inequality.

Our fourth and final series attempts to approximate us, if only superficially, to the differences in nutritional health between urban and rural areas. To do this, we have calculated the proportion of recruits who were excluded from compulsory military service for not reaching the required minimum stature or for suffering from insufficient organic development, that is, physical weakness. This indicator, expressed as a percentage over the total recruits presented for conscription, can be useful to better understand the evolution of adult height, as it captures some of the most obvious consequences of nutritional deficiencies during childhood and youth [51,52,53]. In no case should it be interpreted as a measure of malnutrition in the strict sense.

Regarding the statistical methods used in our analysis, in addition to the average or mean and the 50th percentile (P_50_), both well known, we have used the test of equality of means and the coefficient of variation.

The test of equality of means or T-test for independent samples is a procedure that allows to know if the difference between the means of the different groups that contains a variable is statistically significant or is the result of chance. That is, it allows us to know, for example, if the difference between the average stature of recruits living in urban areas and the average stature of those residing in rural areas is statistically significant or random. The test, carried out in our case through the SPSS program, implies a hypothesis test where the null hypothesis (*H*_0_) is that the difference between the compared means is not statistically significant. To interpret the results obtained by SPSS, we must use the criterion “observed significance level” (sig.). If this value (*p*-value = *α*) is less than the significance level at which the test is performed (normally 5%), we must reject the null hypothesis. On the contrary, if said value is greater than the significance level (*α* = 0.05), there are not enough elements in the sample to reject the null hypothesis.

For its part, the coefficient of variation is the most used procedure to measure anthropometric inequality [8]. Its formula expresses the standard deviation (*σ*) as a percentage of the arithmetic mean (*μ*):

(1) $$\mathrm{CV}=\frac{\sigma }{\mu }\;100$$

The result is easy to interpret: the higher the value of the coefficient, the greater the heterogeneity within the variable, and vice versa.

Both for this coefficient and for the rest of the statistics calculated here, we graphically represent the results by birth cohorts and by recruitment cohorts. Given that at age 21 the effects of the environment (social, economic, nutritional, sanitary, etc.) on the height are clearly in decline, the specialized literature tends to use the date of birth as a reference for the interpretation of anthropometric series. In the graphic representation of our sample, we follow the same criterion, but adding a secondary horizontal axis with the recruitment cohorts. The objective of this axis is to try to capture the circumstances that may have influenced adult height throughout adolescence [54].

## 3. Results

**Figure 3:**
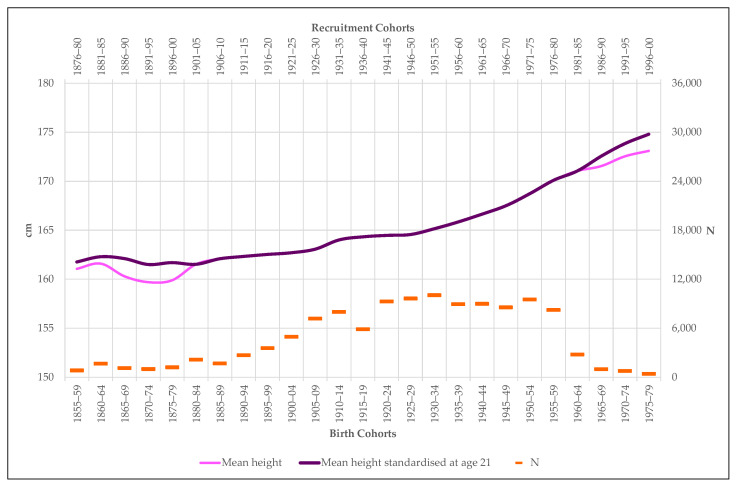
Number (N) and mean height (cm) of recruits included in the sample Extremadura.

Once standardized, the series of statures built for Extremadura is easy to interpret (Figure 3). After four decades of anthropometric stagnation, the male population of Extremadura begins a practically uninterrupted growth process that raises the height difference between recruits born in 1855 and those conscripted in 2000 to just over 13 cm. It is clear that in terms of biological standard of living, measured through adult stature, the end of the 19th century represents for the region the beginning of the “great growth spur” [55]. In the following section we will briefly point out the reasons for this stretching and we will study the only two critical cycles that the series Extremadura’35 presents: the period between 1855 and 1884, associated with the end-of-the-century agrarian crisis, and the period between 1920 and 1929, related to the First World War (1914–1918), the Spanish Civil War (1936–1939) and the postwar period (1939–1951).

**Figure 4:**
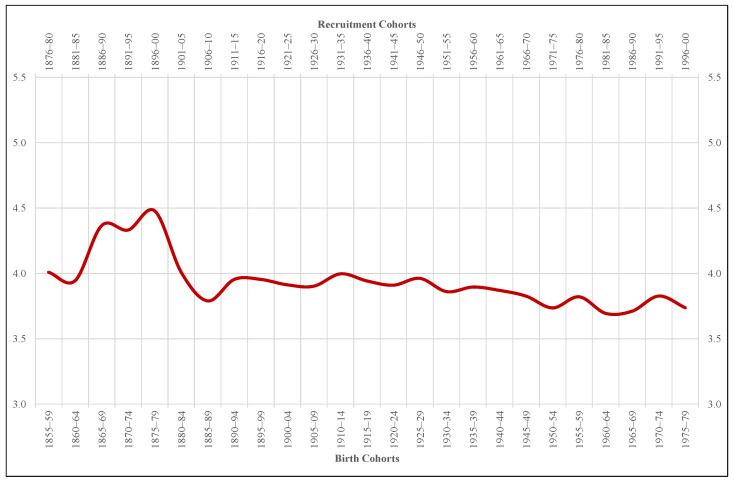
Variation coefficients of the height sample Extremadura (%).

For the moment, we stop in height inequality. From this perspective, the evolution of the variation coefficients (Figure 4) reveals that nutritional heterogeneity increases between 1860 and 1879, coinciding with the end-of-the-century agrarian crisis, but not during the 1920s. In fact, since the end of the 19th century, statistical dispersion tends to stagnation and, from 1925–1929 onward, to decrease. In no case the variation coefficients that our sample throws, never situated above 4.5%, are expressive of an authentic and dramatic nutritional polarization in Extremadura society.

**Figure 5:**
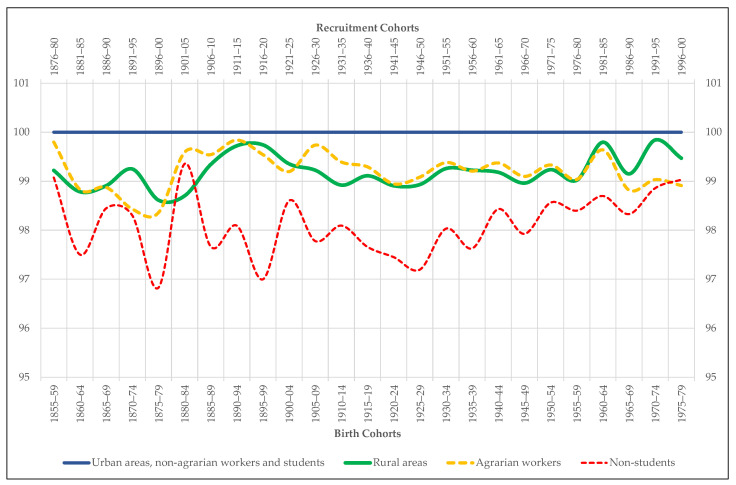
Mean height of the recruits included in the sample Extremadura by place of residence, occupation, and socio-educational status (urban areas, non-agrarian workers and students = 100).

This image of relative equality contrasts with the one shown by the comparison between the height of men from urban areas and that of those living in rural ones (Figure 5). From the contrast we deduce two major trends. On the one hand, we observe that the stature of the conscripts recruited in cities and big towns is always higher than that of those in villages and small localities. The average difference is 1.3 cm, although it exceeds 2 cm between 1875 and 1884. On the other hand, we discover that this difference increases not only throughout the end-of-the-century agrarian crisis, but also during the 1920s, the decade in which the negative effects inherited from the First World War converges with the perverse consequences of the Spanish Civil War and the postwar period. In short, it is the rural world that most intensely suffers the impact of the two great anthropometric crises that Extremadura experiences since the mid-19th century.

The test of equality of means carried out for our sample of statures as a whole reinforces the strength of the urban-rural height gap (Table 2). With an observed level of significance of 0.000, we must reject the null hypothesis and conclude that the difference between the mean height of the conscripts recruited in cities and large towns and the average stature of the recruits measured in villages and small towns is statistically significant. It is therefore clear that it is the rural height penalty hypothesis and not the rural premium one that works in Extremadura throughout the period under study. The rural penalty is, moreover, confirmed when the T-test is applied to each of the cohorts into which we have divided the recruits included in the sample Extremadura’35. Only in the cohorts born between 1890 and 1899 and in those born in 1960–1964, just when the difference in stature is smaller, the test of equality of means offers significance levels higher than 0.05. That is to say, unlike other regions of Spain where urban penalty gives way to rural penalization during the transit between the 19th and 20th centuries, in Extremadura there have never been an urban punishment. There has always been a clear rural height penalty.

The result of our analysis cannot surprise us. We must not forget that we are talking about one of the poorest areas in Europe and, without a doubt, the least industrialized region of Spain. It is true that, according to the occupation data offered by the ACDSs for Extremadura’s recruits, there are visible differences between rural and urban areas in terms of economic structure (Figure 2). Thus, even though the agrarian sector predominates in both areas, it is in large towns and cities where industry acquire a higher proportion. When we talk about industry in Extremadura, however, we are talking mainly about agri-food industry and, in general, by a dispersed and disintegrated activity, incapable of generating zones of manufacturing concentration powerful enough to allow the exploitation of external economies [34]. In other words, we mean an industry that has not stimulated massive migratory movements from the countryside to the city within the region and that it has not generated problems of overcrowding, pollution, or unsanitary conditions in urban areas. Consequently, the environmental differences between urban and rural world in Extremadura have not been in the past nor in the present considerable. Why then are there statistically significant height differences between them?

**Figure 6:**
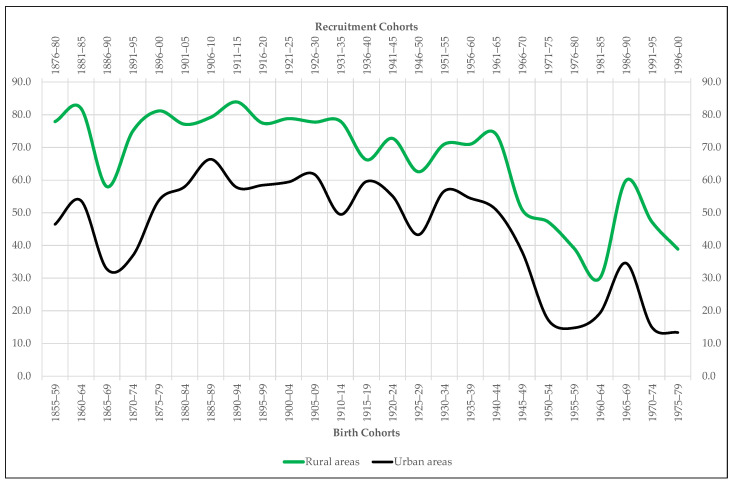
Proportion of recruits included in the sample Extremadura who declare working in the agrarian sector (percentage over the total agrarian and non-agrarian recruits).

To try to answer this question, we resort to the complementary variables extracted from the sample Extremadura’35, starting with the one that distinguishes the stature of agrarian workers from that of non-agrarian workers. The hypothesis to be tested is twofold. On the one hand, we intuit that, given the energy expenditure required by countryside tasks, agrarian workers may have generally been shorter than non-agrarian workers, especially in poor areas with low levels of food consumption. On the other hand, we suspect that agrarian workers, that is, the shortest men according to the previous conjecture, are more concentrated in villages and small towns than in cities and large towns. The results of our tests fully confirm such hypothesis. Figure 5 shows that the height gap between agrarian and non-agrarian workers remains active throughout the entire period under study in favor, of course, of the non-agrarian workers. The test of equality of means (Table 3) confirms that this difference is also statistically significant. In addition, the differences in stature between agrarian and non-agrarian workers follows practically the same evolutionary pattern as the urban-rural gap (Figure 5). For its part, the percentage of agrarian workers residing in villages and small towns is always higher than the proportion of agrarian workers living in cities and large towns (Figure 6). So that, the first conclusion that emerges from our analysis is that in Extremadura the professional gap is probably one of the main determinants of the existing anthropometric gap between the countryside and the city.

**Figure 7:**
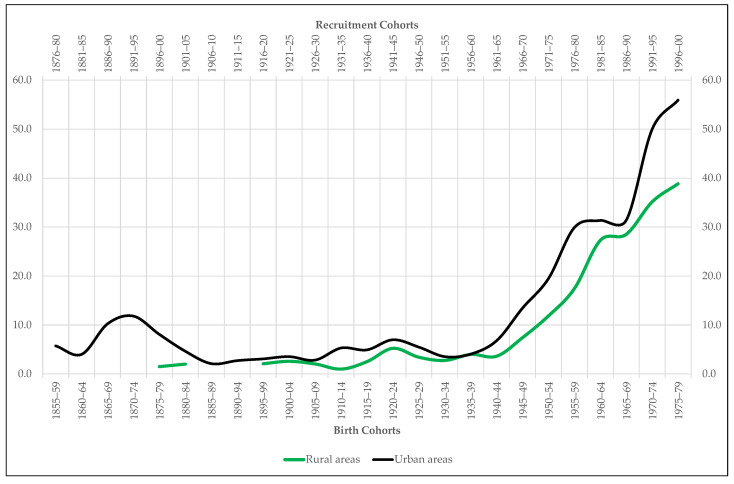
Proportion of recruits included in the sample Extremadura who declare being students (percentage over the total recruits who declare to exercise a profession).

Occupation is not the only variable that could have marked the anthropometric difference between urban and rural areas. For Spain, some studies suggest that while the highest-income social groups are concentrated in urban nuclei, the lowest income groups predominate in rural localities [29]. In Extremadura, this difference also seems to be true if we use the student population as a proxy variable for social status. In this sense, our analysis first reveals that, as in the rest of Spain, students are the tallest of all the young people conscripted in Extremadura from the mid-19th century to the end of the 20th century (Figure 5). The average difference in stature between them and the rest of the recruits who declare to exercise a profession throughout the period under study is 3.2 cm, reaching the 5.3 cm at sometimes, such as 1896–1900. This difference in height is also, of course, statistically significant (Table 4). On the other hand, the proportion of students in urban areas is always higher than that in rural areas (Figure 7). The greater presence of student population in cities and large towns is indicative that social groups with higher incomes and, therefore, with a higher biological standard of living reside there. That is to say, the rural height penalty in Extremadura seems to be associated not only with the greater presence of agrarian workers in villages and small towns, but also with the lower presence of wealthy families in them.

**Figure 8:**
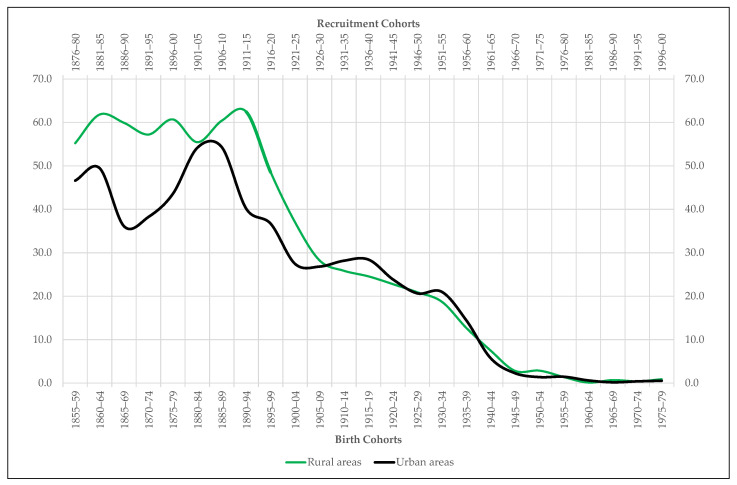
Proportion of recruits included in the sample Extremadura who declare to be illiterate (percentage over the total recruits presented for conscription).

Less conclusive turns out to be the proportion of recruits who claim to be illiterate. In this case, we talk about a variable that not only makes it possible to identify training problems among the recruited population, but also differences in income and access to education among the recruits’ families. In fact, illiteracy continues to be one of the main determinants of malnutrition and poverty in developing countries today [13]. In Extremadura, we know that since the middle of the 19th century, illiterate recruits were always shorter than literate ones [46]. The evolution of illiteracy rates by place of residence, however, does not allow us to speak of the literacy as a determining factor of the urban-rural height gap (Figure 8). It is true that until 1910–1914 these rates are higher in villages and small towns than in cities and big towns. However, since 1890–1894 illiteracy rates fall in both areas, even though the urban-rural height gap remains active until the end of the period under study.

**Figure 9:**
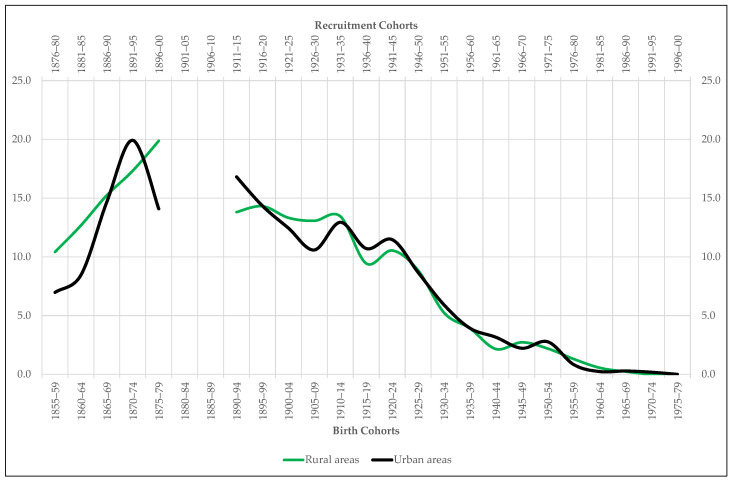
Proportion of recruits included in the sample Extremadura who were excluded from the military service for being short or suffering from insufficient organic development (percentage of total recruits presented for conscription).

Nor does the proportion of conscripts excluded from military service for being short or suffering insufficient organic development seems to be of much importance in understanding the urban-rural height gap. Conceived as a proxy for nutritional health, the evolution of this variable over time does not allow the identification of significant differences between urban and rural areas (Figure 9). Since 1890–1894, in addition, the proportion of recruits excluded from military service due to their physical weakness shows a clear downward trend that makes it impossible to consider it as an explanatory variable for the urban-rural height gap.

## 4. Discussion

In this article, we have studied trends in biological living standard in one of the poorest regions of Spain (Extremadura), delving into the height gap that anthropometric historiography has observed between urban and rural areas. To this purpose, we have worked on a large sample of male statures (Extremadura’35) extracted from the sources of military recruitment (ACDSs) conserved in thirty-five Extremadura’s municipalities. The sample comprises the period between 1855 (year of birth of the first recruits in the study) and 2000 (year in which the last recruits of the sample were conscripted). Given that the Spanish military legislation modified the legal age of enlistment several times and given that, according to the experts, the end of the physical growth of the male population can be delayed until after the age of 20, we have standardized our sample of statures at 21 years. To explore the inequality that it contains, we have calculated coefficients of variation, we have made tests of equality of means, and we have built several complementary time series of economic or professional, social, educational, or nutritional health nature.

Regarding long-term trends, the results of the analysis leave no room for doubt. Once the end-of-the-century agrarian crisis had been overcome, the male population of Extremadura began a cycle of physical growth that, except during the 1920s, remained practically constant until the last decades of the 20th century. For Extremadura, therefore, as for other regions of Spain, the last decades of the 19th century marked the beginning of what some specialists call “the great leap forward” [43].

It is difficult to know the specific reasons for this prolonged stretching. Given the wide margin of physical growth of the Extremadura’s young men born in the last third of the 19th century [56] and in the first decades of the 20th century [57], any small change in the socioeconomic environment could generate large changes in the average height. We believe however that, in general, the intensification of land use since the beginning of the 20th century, the expansion of the sanitation infrastructures and health services from the 1920s, modernization of irrigated agriculture and the electrification of the agri-food industry during the 1950s and, of course, the steady improvement in per capita income in the region were decisive in such leap forward [55,58]. Above all, as has already been studied, the key to anthropometric success in Extremadura, as in the rest of the country, seems to have been associated with the culmination of two well-known parallel processes: the “epidemiological transition” and the “nutritional transition” [59].

In line with other studies, our work also reveals two critical moments for long-term physical growth in Extremadura: the period between 1855 and 1884 and the environmental context related to the First World War, the Spanish Civil War, and the post-war period, which fundamentally affected recruits born in the 1920s. In both cases, the evolution of the mean height of the recruits conscripted in the region shows a clear trend towards stagnation, with annual growth rates of −0.01 and 0.01, respectively.

For the first nutritional bump, the most convincing explanation is related to the end-of-the-century agrarian crisis, caused by the massive arrival in Europe of primary products from overseas [60]. The crisis intensely affected the Extremadura’s economy, specialized in two of the products most affected by the fall in prices to which the “first globalization” gave rise: wool and cereal. In these circumstances, the bad harvests of 1867–1868, 1874, 1879 and 1882, as well as the successive locust plagues suffered in the region [61], contributed to the reduction of the cultivated area, the consequent fall of agricultural production and the reappearance of subsistence crises, characterized by the rapid rise in prices of basic foods [62]. In short, a favorable scenario for maintaining a low nutritional level and, therefore, for anthropometric stagnation.

In the explanation of the second nutritional crisis, less visible in Extremadura than in other areas of Spain, two adverse circumstances converge: the inflation inherited from the First World War and the scarcity derived from the Spanish Civil War and the postwar period. In the first case, the sharp rise in the general price level, especially that of basic foods such as bread, was linked to the rapid expansion of demand by the countries that, unlike Spain, participated in the conflict. The consequent possibility of exporting food at a very good price brought with it in some areas, such as Extremadura, an unusual situation of shortage that raised inflation rates and reduced the purchasing power of the region’s population until well into the 1920s [58].

Young people born in these circumstances not only suffered from nutritional problems during childhood, but also reached puberty amid the severe deprivation resulting from the Spanish Civil War and, especially, from the autarchic policies of the 1940s, the so called “years of hunger” [63]. The reinforcement of the intervention in agricultural production during these years stimulated shortages, prolonging the misery inherited from the war. With a military conception of the markets, the Franco regime tried to “discipline” the process of price formation, closing the borders and forcing producers to sell national production at a maximum price to the State. The political fixation of prices without considering the evolution of costs eliminated the incentives to produce and helped to divert much of what was produced towards smuggling or to *estraperlo*, a market parallel to the official one in which the regulated products reached exorbitant prices [64]. Hence the shortage during the years of hunger, and hence the need to complete the regulation of prices with restrictions on consumption through “ration cards” [65].

It goes without saying that this combination of perverse circumstances led to a worsening of the nutritional levels of the Extremadura’s society, also, of course, of the recruits who lived the adolescent growth during the decades of 1930 and 1940 [66,67]. The harshness of the context in which these young people grew up was accompanied in Spain by a high prevalence of deficiency diseases, such as avitaminosis and pulmonary tuberculosis [68], or of illness related to food hygiene and the water cycles, such as typhoid fever and enteritis [69]. Again, therefore, the pressure of the disease, together with malnutrition, slowed down the process of physical growth that the male population of Extremadura had been experiencing since the last decades of the 19th century.

In terms of inequality, measured through the coefficient of variation, neither the Spanish Civil War nor the postwar period seem to have left too deep a mark on the region. In fact, not even during the end-of-the-century agrarian crisis, a stage in which the statistical dispersion soared, did height inequality reach significant levels in Extremadura. It is more, once the agrarian crisis was ended, the internal heterogeneity of our height sample tended to stagnate and, later, to decline. From this perspective, it should be noted that, unlike some areas of Spain [44] but in correspondence with others [33], in Extremadura the inverse relationship that showed the anthropometric inequality (coefficient of variation) with respect to physical growth (average stature) during the first globalization did not lose consistency in the course of the 20th century.

Nor did the urban-rural height gap lose intensity throughout the period under study. In this regard, our analysis reveals, on the one hand, that the conscripts recruited in urban areas were always taller than those measured in rural ones, and, on the other side, that the anthropometric differences between them were always statistically significant. It is clear, therefore, that the urban penalty hypothesis does not work in our height sample. In fact, unlike some other regions in which the urban penalty was replaced by an urban premium since the last decades of the 19th century, the rural punishment in stature was always the predominant trend in Extremadura. Let us see briefly what reasons are behind this regional difference.

Regardless of the changes in perspective that the rapid advance of anthropometric research has generated in Spain [13], today it seems clear that the urban-rural height gap in the country has historically been more complex than what it has been thought up to now [44]. Some trends, however, do seem clear. The few territories in which there were hints of urban penalization during the 19th and 20th century were those in which sooner or later the first industrialization took hold, and/or in which the agrarian sector made it possible to maintain high levels of nutritional well-being. In the first case (Bilbao, Igualada, Reus, Elche, or Alcoy, for example), the harsh working conditions and the use of child labor in factories and mines, the rapid population growth, and the consequent overcrowding, as well as environmental and epidemiological degradation of cities, seem to have created the most suitable context for the deterioration of biological living standards, at least temporarily [44,70,71,72,73,74]. In the second but not excluding case (Basque Country or Valencia), the better distribution of land ownership, the greater agrarian specialization, and/or the technical improvement allowed to sustain in the countryside a level of nutrition higher than those registered in cities, thus guaranteeing the rural premium [33,55,75].

In these circumstances, the case of Extremadura is easy to explain. On the one hand, it should be remembered that the Industrial Revolution passed almost on tiptoe through Extremadura [76]. Only since the 1940s, first taking advantage of the business opportunities generated by the postwar black market and, later, the increase in public investment in the region, some municipalities, above all medium-sized towns, began a timid industrialization process. In no case did this process succeed in increasing the size of cities, creating economies of scale, or modernizing the sector. If anything, it contributed to strengthening the traditional agri-food industry. Not even during the period of developmentalism (1964–1974), the years of the “Spanish economic miracle”, did the Extremadura’s industry manage to make an important productive leap [77]. It continued without generating employment and kept contributing a truly small share to the regional GDP, thus consolidating that “manufacturing desert” that began to forge in the late 18th century [78].

If on the industrial side it was difficult to suffer urban height penalty in Extremadura, on the agriculture side it was also practically impossible to enjoy a rural premium. With almost more intensity than in the rest of Spain, the predominance of large estates in the region and the concentration of land ownership in very few hands have historically been the main characteristics of Extremadura’s agriculture [79]. These two characteristics converge in the so-called system of *dehesa*, a model of agrosilvopastoral exploitation dominated by extensive cattle ranching and rainfed agriculture [80]. Singularized by its productive versatility, but also by its low productivity, the dehesa has traditionally favored the proletarianization of the peasant family and has limited the possibility of significantly increasing the income of most of the rural population. On the other hand, the technical improvement of the agrarian sector has not reached Extremadura until the mid-1950s, coinciding with the greater public investment in the region, mainly in irrigation infrastructures, and with the cheapening of the technologies of the green revolution. In such a situation, of course, it has been impossible for regional agriculture to raise the biological standard of living in rural areas above that of urban ones.

It should be pointed out on this regard that in Extremadura, as in the rest of deindustrialized Spain, the green revolution rapidly raised the productivity of labor in the farms in the region. The consequent departure of surplus labor abroad (France, Switzerland, and Germany) or to other areas of the country (Catalonia, the Basque Country, Navarre, and Valencia, mainly) originated a process of “artificial biological selection” that left in the rural world to the weakest part, anthropometrically speaking, of the male rural population [36]. The migratory hemorrhage did not generate overcrowding or unhealthy conditions in the urban areas of Extremadura, but instead diverted demographic pressure to the most industrialized cities of Europe. In this way, the crisis of traditional agriculture in the region from the mid-1950s onwards not only did not contribute to feed a late urban height punishment, but also triggered a process of internal differentiation in the region that kept the rural height penalty active.

Other factors, in addition to the agrarian and industrial backwardness, can explain the forceful of this rural height penalization. As in many other areas of Spain, Extremadura cities were not only less industrialized and less populated than those of other European regions, but they were mostly residential and administrative areas. In them lived the social elites, the emerging middle classes and the bulk of industrial workers, artisans, merchants, and service sector personnel. Definitely, a whole range of social and professional groups that normally had family income that maximized the quality of nutrition [13]. In most cities and large towns there were also greater possibilities of access to educational, care and health services [56]. It is more, vaccination, food and work hygiene controls were more efficient in the city than in the countryside [81]. Some studies also indicate that urban areas offered greater possibilities in the field of nutrition, since they had efficient systems of food storage, conservation, and distribution [75].

On the contrary, in rural areas there were high levels of poverty and malnutrition. It is true that the rural population enjoyed some advantages in terms of biological well-being by living in a more favorable environment and having easier access to sources of nutrients. However, a large part of the people who lived in the countryside remained oblivious to the provision of basic infrastructures, such as health or education, until well into the 20th century. In fact, the Spanish rural population was not covered by health public services until the 1960s [82]. On the other hand, although landowners and the largest rentiers of the land resided in the cities and big towns, in the rural world lived mainly groups associated with agriculture and livestock, with a predominance of day laborers, peasants, farmers, shepherds and small owners of land and livestock [13]. Ultimately, the urban-rural height gap did not depend on the size of the place of residence, but on the economic, social, educational, health, institutional and environmental possibilities that the place of residence offered.

In our study we have delved only into those factors for which the ACDSs offer reliable information: economic, social, educational, and health factors. From an economic perspective, we have used the data offered by this source on the occupation of the recruits. Starting from the idea that the greater physical effort traditionally required by the agricultural and livestock tasks makes agrarian workers shorter than non-agrarian workers, we have compared the average stature of these two groups of professionals. The results of the comparison are quite conclusive. The anthropometric difference between ones and the others is maintained throughout the entire period under study and is also statistically significant. Therefore, and given that the countryside hosts a higher proportion of agrarian workers than the city during the period under study, it can be concluded that an important part of the rural height penalty that persists in Extremadura since the mid-19th century is due to the anthropometric penalization suffered agrarian workers with respect to non-agrarian ones.

From a social perspective, the hypothesis to be tested is that the higher-income groups and, therefore, those with greater access to nutrition, education, and health preferably live-in urban areas. Among the little information that the ACDS offers in this regard is that which distinguishes the students from the rest of the recruits who declare to exercise a profession. It should be remembered that when we speak of students, we are speaking mainly about university students. For their families, this situation historically implied a high social am economic level. Not only because, while a young man was studying, he did not contribute income to the family, but because until the extension of public grants during the last decades of the 20th century the expenses of a student could be truly considerable. Let is think not so much in terms of study material, but above all in terms of accommodation costs. It should be considered that high schools were preferably located in urban areas and that until 1973 there was no university in Extremadura, so young people who wanted and could study a university degree had to do so outside the region, generally in Madrid, Salamanca, or Seville.

Therefore, if we understand the student population as a proxy variable of social status, we must conclude that the hypothesis that links the rural height penalty with a lower presence in rural areas of social elites seems to be true in Extremadura. Not by chance, our analysis confirms that the students were by far the tallest of all the young men recruited in the region since the mid-19th century. The research also reveals that the proportion of students living in urban areas was not only higher than that of students living in rural ones, but that this difference increased after the Spanish Civil War. From this perspective, the importance of social inequality as a determining factor of the urban-rural height gap in Extremadura acquires a dimension that other proxy variables such as the illiteracy rate prevent us from perceiving. When looking for the causes that explain why men living in rural Extremadura were always shorter than urban dwellers, we must focus mainly on profession and on social status.

Different is conclusion raised by the information that the ACDSs offer on alphabetization, information that, very cautiously, can be also considered as a proxy variable of socioeconomic status. It should be noted in this sense that literacy can be understood as the ability to access knowledge [83], a definition that fits well with the hypothesis that the education of parents and children can mark differences in the stature reached at the end of the stage of physical growth [48]. In the case of Extremadura, it seems clear that the average stature of illiterate recruits was always lower than that of literate conscripts [46]. What is not clear is that this difference can explain the rural height penalty. In this sense, our analysis reveals that, during the second half of the 19th century, the percentage of illiterates was higher in the countryside than in the city. Since the late 19th and early 20th centuries, however, illiteracy rates began to plummet and the differences by place of residence tended to dilute. These rates, therefore, cannot explain why the urban-rural height gap continued to exist in Extremadura until the end of the 20th century.

And what about nutritional health? Is it not a decisive factor for physical growth and, therefore, to understand the differences in stature observed between urban and rural areas? The specialized literature maintains that it is, but our data do not allow us to confirm it. Such data, prepared from the information offered by the ACDSs on the recruits who were excluded from compulsory military service for being short in stature or for suffering from insufficient organic development, confirm the nutritional deficiencies of the last decades of the 19th century in Extremadura, but they do not mark significant differences between urban and rural areas. We must remember in any case that these data cannot be considered indicators of malnutrition in the strict sense. Therefore, it is advisable to be careful when trying to weigh the incidence of nutritional health over the urban-rural height gap with proxy variables that may be debatable.

From this perspective, one last methodological consideration must be made. With the sources of military recruitment in hand, it is more than tempting to try to build other more powerful health indicators. It should be noted in this regard that the ACDSs also provide individualized information on illnesses or physical problems that recruits claim to suffer in order to be excluded from compulsory military service. These diseases range from mental disorders to bone problems, through pathologies related to sight, ears, stomach, intestines, and a long etcetera. So, what is the problem? The problem is that very few times, diseases that are related to nutrition or that have clear effects on physical growth are indicated in the ACDSs. Once again, therefore, prudence must be imposed while waiting for specific epidemiological studies to allow us to know whether rural height penalty obey a state of health inferior to that of the urban world.

## 5. Conclusions

Starting from the idea that adult stature is a good indicator of the biological standard of living, this article delves into nutritional inequality, more specifically in the open debate within anthropometric historiography on the urban-rural height gap. For this, the present study uses a wide sample of statures extracted from the sources of military recruitment conserved in thirty-five municipalities of Extremadura, one of the poorest regions in Europe and the least industrialized of all Spain. The height sample, called Extremadura’35, comprises the period between 1855 (year of birth of the first recruits of the sample) and 2000 (year of conscription of the last recruits that our sample of statures includes). It is thus situated in the middle of the broad spectrum of improvements that have accompanied to contemporary social and economic modernization, among them, of course, the epidemiological and nutritional transition.

The combination of these processes of change, together with the slow but important socioeconomic improvements experienced by Extremadura since the last decades of the 19th century, explains the powerful physical growth registered by the male population conscripted in the region during the period under study. Only two critical moments seem to have slowed down this spectacular increase in adult stature: the period between 1855 and 1884 and the period between 1920 and 1929. The first of them is defined in Extremadura by the scarcity generated by the last subsistence crises of the 19th century, especially those caused by the so-called end-of-the-century agrarian crisis. In the second period, less dramatic in anthropometric terms than in other regions of Spain, the nutritional deficiencies inherited from the First World War converge with the deprivations suffered by those who go through the adolescent growth during the Spanish Civil War and the postwar period.

Only for the first of the two indicated moments, our study reveals a substantial increase in height inequality, at least as measured by the variation coefficients and considering the small margin of heterogeneity shown by such coefficients for Extremadura. From this perspective, the conclusion derived from the present research is that in the long term there is an inverse relationship between physical growth and anthropometric inequality. In other words, the greater the growth in stature, the less height inequality. This general conclusion contrasts with those extracted from other studies carried out in Spain, but in no case does it deny the existence of an anthropometric gap between urban areas (cities and large towns) and rural ones (villages and small towns).

From this other way of analyzing nutritional inequality, our study clearly demonstrates that it was the rural height penalty and not the urban penalization that predominated in Extremadura from the mid-19th century to the late 20th century. It is more, the analysis carried out here confirms that it was the population living in villages and small towns in the region that suffered most intensely the severe consequences of the end-of-the-century agrarian crisis and, to a lesser extent, the effects of the First World War, the Spanish Civil War, and the postwar period.

There are many factors that can explain this rural punishment, both in Extremadura and in most of Spain. On the urban side, it is worth highlighting: the lower overcrowding and pollution of Spanish cities compared to the more industrialized countries, the greater presence in such areas of the higher-income social and professional groups, the better access to educational and health services and, even, the existence of more efficient systems of conservation and distribution of food. On the rural side, no one denies some advantages, such as greater environmental health and easier and faster access to sources of nutrients. More important, however, are the disadvantages with respect to the urban world, especially the difficulty of access to basic health, education, or welfare infrastructures, as well as the preponderance of the agrarian population, generally of lower income and forced to develop a greater physical effort than that of the non-agrarian population.

In the case of Extremadura, there are also specific historical conditions that reinforce the rural penalization in terms of the biological well-being. Not in vain do we speak of a never industrialized region that not only did not generate massive exoduses from the countryside to the city within the region, but additionally fueled strong migratory movements to other territories since the mid-1950s. Until then, in addition, the agrarian sector was characterized in Extremadura by limited technical improvement and very low productivity. This lack of innovation, together with a highly concentrated distribution of land ownership, has historically favored the proletarianization of the peasant family and the persistence of low per capita income. We are therefore faced with an economic, technical, and institutional structure incapable of substantially raising the nutrition levels of most of the rural population.

With this background, our research delves into the determinants of the rural height penalty that the sources of military recruitment show for Extremadura. These same sources seem to contain two of the explanatory keys for such penalization: the professional structure and the social composition of the villages and small towns in the region. Regarding the professional structure, our study confirms the hypothesis that, in areas with a low nutritional level, like Extremadura, the physical effort required by the tasks of the agriculture and livestock keeps the average statures of the agrarian population below the mean of the non-agrarian population. In the same way, this research reveals that, despite the importance that the agrarian population acquires in the so-called agro-cities, in Extremadura it is in rural areas where a greater proportion of agrarian workers are concentrated. Despite the progressive loss of importance suffered by farming activity in the region since the beginning of the 20th century, this proportional difference is maintained throughout the period under study. Everything therefore seems to indicate that one of the reasons that explain the persistence of the urban-rural height gap in Extremadura since the mid-19th century is the prevalence in rural areas of a labor (economic) structure that generates shorter statures than those of urban areas.

Concerning the social composition of villages and small towns, we only have indirect information from the sources of military recruitment. The best data on this regard are the ones that makes it possible to distinguish students, generally university ones, from among recruits who declare exercising a certain profession. Studying a university career in Spain has historically been a luxury available to very few families. In fact, all the investigations carried out so far in the country coincide in pointing out the students as the tallest among the conscripted population. This is an evident proof that students have traditionally belonged to the families with the highest incomes and, consequently, with better access to nutrition, hygiene, and health. In the specific case of Extremadura, our research reveals that students were the highest of all recruits conscripted in the region since the last decades of the 19th century. The article additionally reveals that the proportion of students residing in rural areas was also always lower than that in urban areas. The conclusion, therefore, seems clear: the lower presence of the more affluent families of society in villages and small towns is another reason that explains the persistence of the rural height penalty in Extremadura since the mid-19th century.

The social factor does not exhaust the list of factors that can explain the existing nutritional gap in Extremadura between the countryside and the city. The problem is that the sources of military recruitment do not allow us to go much further. It is true that these sources do contain enough information to test, for example, the incidence of education, more specifically literacy, in this urban-rural height gap. In fact, previous research has shown that literate recruits have always been taller than non-literate ones. In the present article we have tried to go a little further by measuring the proportion of illiterates in urban and rural areas. The results, however, are not conclusive. According to them, there are only significant differences between one area and other until the beginning of the 20th century. From the mid-1880s, however, the proportion of illiterates residing in both urban and rural areas has not stopped falling. Given that in the meantime the urban-rural height gap has remained active, it is difficult to argue that the educational factor is a determining factor in the persistence of such a gap.

The same is true of the variable used here as an alternative measure of nutritional health: the proportion of recruits who were excluded from military service for being short or suffering physical weakness. Expressed as a percentage of the total number of recruits presented in each recognition act and disaggregated down by place of residence, this proxy variable draws a trajectory that is hardly compatible with the rural height penalty experienced by Extremadura since the mid-19th century. First, because it does not allow to identify significant differences between cities and large towns on the one hand and villages and small towns on the other. And, secondly, because since the mid-1890s this proxy variable begins a sustained decline in both areas that ends up dissolving over time despite the persistence of the rural height punishment.

In short, our study confirms the relevance of economic and social factors for the analysis of rural penalization. It also reveals that the sources of military recruitment do not allow to ratify the importance of education or nutritional health in the study of such penalization. In no case does our research attempt to deny the, on the other hand, demonstrated incidence of both variables in the evolution of the biological standard of living. What it denies is the possibility of considering them as determining factors of the urban-rural height gap that Extremadura, like so many other regions of Spain, has suffered since at least the middle of the 19th century.

## Figures and Tables

**Table 1:** Increase in the mean age of the recruits included in the sample Extremadura (1855–1890 and 1954–1982).

Years Old	Birth Years	P_50_	Increase	
			Recruitment Age	Centimeter
19	1866–1880	160.1	19–20	1.0
20	1858–1864 and 1881–1885	161.1	20–21	0.8
21	1855–1856 and 1886–1890	161.9	19–21	1.8
18	1978–1982	169.3	18–19	0.8
19	1972–1976	170.1	19–20	0.6
20	1966–1970	170.7	20–21	0.3
21	1954–1964	171.0	19–21	0.9
			18–21	1.7

SOURCES: Own elaboration based on the ACDSs of Aceuchal, Almendralejo, Azuaga, Arroyo de la Luz, Barcarrota, Cáceres, Caminomorisco, Campo Lugar, Coria, Don Benito, Fuentes de León, Garrovillas de Alconétar, Hervás, Jaraíz de la Vera, Jerez de los Caballeros, La Albuera, La Coronada, Madroñera, Magacela, Mérida, Montánchez, Plasencia, Oliva de la Frontera, Quintana de la Serena, Salvaleón, San Vicente de Alcántara, Serradilla, Valle de la Serena, Valverde de Leganés, Villafranca de los Barros, Villanueva de la Serena, Zafra, Zahínos, Zarza la Mayor, and Zorita.

**Table 2:** T-test for equality of means in the sample Extremadura (urban and rural areas).

		Levene’s Test for Equality of Variances		T-Test for Equality of Means						
		F	Sig.	t	df	Sig.	Means Diff.	Std. Error Diff.	95% Confidence Interval of the Difference	
									Lower	Upper
Height (cm)	Equal variances assumed	0.011	0.918	32.37	120,077	0.000	1.344	0.041	1.262	1.425
	Equal variances not assumed			32.33	97,321	0.000	1.344	0.041	1.262	1.425

**Table 3:** T-test for equality of means in the sample Extremadura (agrarian and non-agrarian workers).

		Levene’s Test for Equality of Variances		T-Test for Equality of Means						
		F	Sig.	t	df	Sig.	Means Diff.	Std. Error Diff.	95% Confidence Interval of the Difference	
									Lower	Upper
Height (cm)	Equal variances assumed	67.61	0.000	−38.07	46,925	0.000	−2.422	0.063	−2.547	−2.297
	Equal variances not assumed			−38.01	46,296	0.000	−2.422	0.063	−2.547	−2.297

**Table 4:** T-test for equality of means in the sample Extremadura (students and non-students).

		Levene’s Test for Equality of Variances		T-Test for Equality of Means						
		F	Sig.	t	df	Sig.	Means Diff.	Std. Error Diff.	95% Confidence Interval of the Difference	
									Lower	Upper
Height (cm)	Equal variances assumed	2.117	0.146	50.24	46,925	0.000	5.569	0.110	5.352	5.786
	Equal variances not assumed			49.76	4957	0.000	5.569	0.111	5.350	5.789
